# Daily Viral Kinetics and Innate and Adaptive Immune Response Assessment in COVID-19: a Case Series

**DOI:** 10.1128/mSphere.00827-20

**Published:** 2020-11-11

**Authors:** Pauline Vetter, Christiane S. Eberhardt, Benjamin Meyer, Paola Andrea Martinez Murillo, Giulia Torriani, Fiona Pigny, Sylvain Lemeille, Samuel Cordey, Florian Laubscher, Diem-Lan Vu, Adrien Calame, Manuel Schibler, Frederique Jacquerioz, Géraldine Blanchard-Rohner, Claire-Anne Siegrist, Laurent Kaiser, Arnaud M. Didierlaurent, Isabella Eckerle

**Affiliations:** a Geneva Centre for Emerging Viral Diseases, Geneva University Hospitals, Geneva, Switzerland; b Division of Infectious Diseases, Geneva University Hospitals, Geneva, Switzerland; c Laboratory of Virology, Division of Laboratory Medicine, Geneva University Hospitals, Geneva, Switzerland; d Faculty of Medicine, University of Geneva, Geneva, Switzerland; e Center for Vaccinology, Department of Pathology and Immunology, Faculty of Medicine, Geneva University Hospitals, Geneva, Switzerland; f Division of General Pediatrics, Geneva University Hospitals, Geneva, Switzerland; g Emory Vaccine Center, Emory University, Atlanta, Georgia, USA; h Department of Microbiology and Molecular Medicine, Faculty of Medicine, University of Geneva, Geneva, Switzerland; U.S. Centers for Disease Control and Prevention

**Keywords:** SARS-CoV-2, cytokines, viral load, immunity, antibody response, COVID-19

## Abstract

Viral shedding patterns and their correlations with immune responses are still poorly characterized in mild coronavirus (CoV) disease 2019 (COVID-19). We monitored shedding of viral RNA and infectious virus and characterized the immune response kinetics of the first five patients quarantined in Geneva, Switzerland. High viral loads and infectious virus shedding were observed from the respiratory tract despite mild symptoms, with isolation of infectious virus and prolonged positivity by reverse transcriptase PCR (RT-PCR) until days 7 and 19 after symptom onset, respectively. Robust innate responses characterized by increases in activated CD14^+^ CD16^+^ monocytes and cytokine responses were observed as early as 2 days after symptom onset. Cellular and humoral severe acute respiratory syndrome (SARS)-CoV-2-specific adaptive responses were detectable in all patients. Infectious virus shedding was limited to the first week after symptom onset. A strong innate response, characterized by mobilization of activated monocytes during the first days of infection and SARS-CoV-2-specific antibodies, was detectable even in patients with mild disease.

**IMPORTANCE** This work is particularly important because it simultaneously assessed the virology, immunology, and clinical presentation of the same subjects, whereas other studies assess these separately. We describe the detailed viral and immune profiles of the first five patients infected by SARS-CoV-2 and quarantined in Geneva, Switzerland. Viral loads peaked at the very beginning of the disease, and infectious virus was shed only during the early acute phase of disease. No infectious virus could be isolated by culture 7 days after onset of symptoms, while viral RNA was still detectable for a prolonged period. Importantly, we saw that all patients, even those with mild symptoms, mount an innate response sufficient for viral control (characterized by early activated cytokines and monocyte responses) and develop specific immunity as well as cellular and humoral SARS-CoV-2-specific adaptive responses, which already begin to decline a few months after the resolution of symptoms.

## Abstract (precis)

This work is particularly important because it simultaneously assessed the virology, immunology, and clinical presentation of the same subjects, whereas other studies assess these separately. We describe the detailed viral and immune profiles of the first five patients infected by SARS-CoV-2 and quarantined in Geneva, Switzerland. Viral loads peaked at the very beginning of the disease, and infectious virus was shed only during the early acute phase of disease. No infectious virus could be isolated by culture 7 days after onset of symptoms, while viral RNA was still detectable for a prolonged period. Importantly, we saw that all patients, even those with mild symptoms, mount an innate response sufficient for viral control (characterized by early activated cytokines and monocyte responses) and develop specific immunity as well as cellular and humoral SARS-CoV-2-specific adaptive responses, which already begin to decline a few months after the resolution of symptoms.

## INTRODUCTION

As of 18 June 2020, more than 8 million cases of coronavirus (CoV) disease 2019 (COVID-19) had been reported worldwide, with 450,000 deaths (1). Although the first reports mainly described patients presenting with severe pneumonia (2), the spectrum of disease is wide, with more than 80% of infected individuals displaying moderate, mild, or even no symptoms (3).

Studies to date have focused on hospitalized patients with severe or critical disease. In those patients, the peak viral load in the upper respiratory tract occurs during the second week post-onset of symptoms (POS), while viral clearance is achieved after 10 days in more than 90% of patients with mild disease (4). Elevated cytokine levels, including levels of interleukin 6 (IL-6) and IP-10, increased C-reactive protein, and profound T-cell lymphopenia signal disease worsening (5–8). This dysregulated inflammatory response is thought to result from an initial impairment of interferon production and thus reduced early viral control. Several reports have suggested that severe acute respiratory syndrome (SARS)-CoV-2 antibody titers are higher in patients with more severe disease (2, 9). Activated SARS-CoV-2-specific CD4 and CD8 T cells have been reported in a few studies (10–12). However, viral shedding and immune responses in patients with mild COVID-19, the most frequent form of disease, remain poorly characterized. Comprehensive studies investigating viral shedding and immune responses at multiple time points for these patients are lacking. Available descriptions and interpretations of viral behavior and immunological responses in more severely ill patients may be biased by the rapid implementation of immunomodulatory treatments.

In Geneva, Switzerland, the first known case of COVID-19 was identified on 26 February 2020. In line with public health policy early in the outbreak, every confirmed patient was hospitalized, including previously healthy young adults with only mild influenza-like illnesses. Here, we report the detailed clinical, virological, and immunological characterization of the first five patients assessed at the Geneva University Hospital (HUG), from the day of diagnosis until convalescence.

## RESULTS

### Clinical characteristics.

Baseline demographic and clinical characteristics of the patients, all adult men, are shown in Table 1. Four patients (P1 to -4) had only mild upper respiratory tract infections (URTI) and required no or only antipyretic treatment. Their mild and unspecific presentation was typical for the majority of COVID-19 patients (sore throat, cough, fever), including P1, who had a very short symptomatic period of only 1 day. The fifth patient (P5) had a severe illness with bilateral pneumonia; computed tomography (CT) revealed bilateral patchy infiltrates involving each pulmonary lobe and a left posterior basal consolidation 7 days POS (see Fig. S1 in the supplemental material) and was included into our study only after 6 days of symptoms. On day 3 of hospitalization, he developed a maculopapular rash on his back and trunk. His treatment included oxygen support, a 7-day course of antibiotics, and lopinavir/ritonavir (13). He recovered and was discharged after 10 days (day 15 POS). Laboratory parameters and in-depth clinical descriptions are available in Table S1.

**TABLE 1:** Main characteristics of the patients

Patient^*a*^	Age (yr)	Comorbidity(ies)	Duration of illness (days)	Maximal temp (°C)^*b*^	% oxygen saturation, % RA with oxygen (FiO_2_)^*c*^	Symptoms	Diagnostic	Treatment(s)
1	28	None	1	38	99 (RA)	Fever, rhinorrhea, headache	Upper respiratory tract infection	None
2	30	None	5	38.4	99 (RA)	Fever, odynodysphagia, dry cough, headache	Upper respiratory tract infection	Paracetamol
3	55	Enlarged prostate	6	38.5	99 (RA)	Fever, odynodysphagia, dry cough, chest pain	Upper respiratory tract infection	Alfuzosin, paracetamol
4	24	None	9	39	100 (RA)	Fever, fatigue, dry cough	Upper respiratory tract infection	Ibuprofen, paracetamol, enoxaparin
5	66	Enlarged prostate, hip arthrosis, hypotestosteronemia	16	37.3	92 (32)	Fatigue, productive cough, skin rash	Severe bilateral pneumonia	Amoxicillin + clarithromycin, piperacillin/ tazobactam, lopinavir/ritonavir, folic acid, enoxaparin

a All of the patients were male.

b During hospitalization.

c RA, room air; FiO_2_, fraction of inspired oxygen.

10.1128/mSphere.00827-20.2 TABLE S1 Detailed characteristics of the patients. CRP, C reactive protein; PCT, procalcitonin; AST, aspartate aminotransferase; ALT, alanine aminotransferase; GGT, gamma-glutamyl transferase. Download Table S1, DOCX file, 0.01 MB. Copyright © 2020 Vetter et al. 2020 Vetter et al. This content is distributed under the terms of the Creative Commons Attribution 4.0 International license.

10.1128/mSphere.00827-20.4 FIG S1 CT scan of patient 5 showing bilateral patchy ground-glass opacities, mainly in the upper lobes, and posterior-basal consolidation, more marked on the left side. Download FIG S1, TIF file, 1.4 MB. Copyright © 2020 Vetter et al. 2020 Vetter et al. This content is distributed under the terms of the Creative Commons Attribution 4.0 International license.

### Viral shedding patterns.

**FIG 1:**
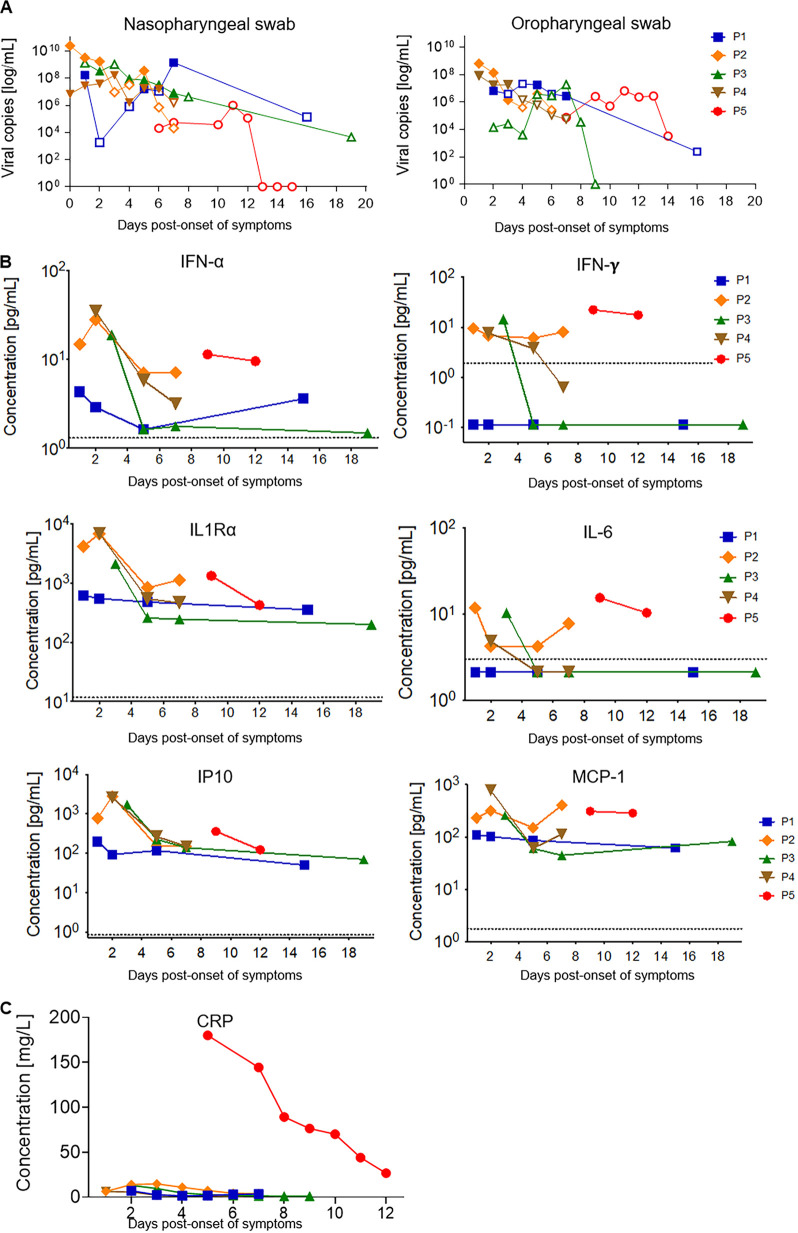
Kinetics of viral load and inflammatory markers. (A) Viral kinetics of nasopharyngeal swabs (NPS) and oropharyngeal swabs (OPS). Filled symbols, isolation of infectious virus in cell culture; open symbols, infectious virus could not be isolated in cell culture. (B) Cytokine and chemokine dynamics. Individual concentrations in picograms per milliliter for each marker were plotted at different days POS for each patient. The dotted line represents the limit of detection for each marker. Samples with undetectable concentrations were arbitrarily given a value of 50% of the last standard dilution value. (C) C-reactive protein (CRP) dynamics. Concentrations in milligram per liter were plotted at different days POS for each patient.

SARS-CoV-2 was detected by real-time reverse transcriptase PCR (rtRT-PCR) in both the oropharyngeal swabs (OPS) and the nasopharyngeal swabs (NPS) of each patient, with high viral loads that declined over time (Fig. 1A and Table S2). Higher viral loads were detected in NPS than in OPS (means of 1.1E9 viral RNA copies/ml and 4.1E7 copies/ml, respectively), with a peak viral load of 2.5E10 copies/ml for P2, sampled at the day of symptom onset. Lower viral loads in NPS than the viral loads of P1 to -4 on days 6 to 7 POS were consistently observed for P5, for whom sampling started only on day 6 POS and covered the second week of illness.

10.1128/mSphere.00827-20.3 TABLE S2 Viral loads retrieved in nasopharyngeal swabs (NPS) and oropharyngeal swabs (OPS). P1 to P5, patients 1 to 5. DPO, days post-onset of symptoms. Download Table S2, XLSX file, 0.01 MB. Copyright © 2020 Vetter et al. 2020 Vetter et al. This content is distributed under the terms of the Creative Commons Attribution 4.0 International license.

Isolation of infectious virus was successful from both NPS and OPS during the first week of illness for the four mild cases, with the last positive virus isolation on day 7 for two patients (P1 and -3). The mean viral load in samples affording successful isolation was 1.2E9 copies/ml; the lowest viral RNA load in a sample from which a positive isolate could be obtained was 1.4E6 copies/ml. No infectious virus could be isolated from any sample from P5.

Prolonged RNA detection was observed in three patients, with persistent positive RT-PCR results on days 14 (P5), 16 (P1), and 19 (P3) POS, albeit with low viral loads. RNA was also detected by RT-PCR in saliva and stool specimens, with peak viral loads of 7.8E6 and 8.6E5 copies/ml, respectively (Fig. S2). No virus was detected in other specimen types, and no RNA was observed in plasma. No virus isolation was attempted from nonrespiratory samples.

10.1128/mSphere.00827-20.5 FIG S2 Viral load in saliva, stool, tears, and urine. Copy numbers of specimens in which SARS-CoV-2 was detected from all 5 patients are shown. Each dot represents a single sample of the respective specimen from a single time point during the course of the collection period. Download FIG S2, TIF file, 0.01 MB. Copyright © 2020 Vetter et al. 2020 Vetter et al. This content is distributed under the terms of the Creative Commons Attribution 4.0 International license.

Full-genome sequences from P1 to -4 were found to cluster with sequences representative of the closest geographically related outbreak at that time, which was in northern Italy. Molecular epidemiology is consistent with histories of travel to the region shortly before symptom onset for P1, P2, and P3 (Fig. S3).

10.1128/mSphere.00827-20.6 FIG S3 Phylogenetic tree constructed on the basis of SARS-CoV-2 complete genome sequences. (A) The evolutionary analyses were conducted in MEGA X using the maximum likelihood method and Hasegawa-Kishino-Yano model. All sequences were downloaded from the GISAID database (for more information, refer to the gisaid_hcov-19_acknowledgement_table below), including four out of the first five patients infected by SARS-CoV-2 and quarantined in Geneva (human CoV [hCoV]-19/Switzerland/GE3895/2020 [P1], hCoV-19/Switzerland/GE9586/2020 [P2], hCoV-19/Switzerland/GE3121/2020 [P3], and hCoV-19/Switzerland/GE0199/2020 [P4]). The scale bar indicates nucleotide substitutions per site. (B) GISAID HcoV-19 acknowledgment table. Download FIG S3, DOCX file, 0.2 MB. Copyright © 2020 Vetter et al. 2020 Vetter et al. This content is distributed under the terms of the Creative Commons Attribution 4.0 International license.

A coinfection with adenovirus at a cycle threshold (Ct) value of 36 was detected in P1 from NPS collected at the time of his diagnosis. No other coinfections were detected in P2 to P5.

### Kinetics of the innate response.

An increase in cytokine levels was observed in the first days POS (Fig. 1B). Patients with mild symptoms had an increase in alpha interferon (IFN-α), IFN-γ, and IP-10 around days 2 to 3, which returned to baseline as symptoms resolved and viral load decreased. IL-6, which has been associated with severe disease (14), was slightly more elevated at early time points, as were IL-1Ra and MCP-1. Their levels were similar to the ones observed in P5 when he was oxygen dependent. Cytokine levels were generally lower in the patient with the mildest symptoms (P1). There were no significant changes in the other cytokines tested, such as TNF-α, IL-1α, IL-2, or IL-8 (not shown). C-reactive protein levels were much higher in P5 than in patients with mild disease (Fig. 1C), suggesting that innate responses can efficiently be triggered in mild disease.

**FIG 2:**
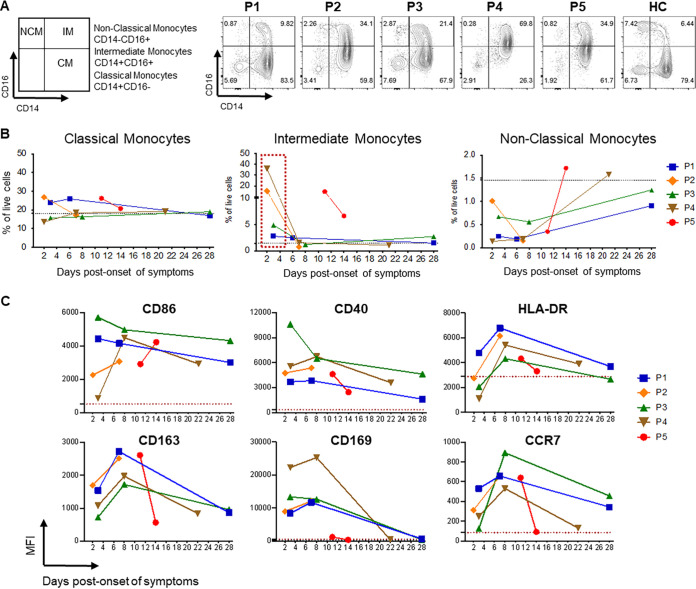
Early increase in intermediate monocytes after SARS-CoV-2 infection. (A) Gating strategy for the identification of different monocyte subsets, classical monocytes (CM), intermediate monocytes (IM), and nonclassical monocytes (NCM), by flow cytometry after gating out dead cells and lineage (CD3, CD20) cells and gating on HLA-DR^+^ cells. Early time point flow cytometry counterplots from P1 to P5 as well as that of a healthy control (HC) are shown. (B) Percentages of CM, IM, and NCM calculated from the lymphocyte live gate for each patient at different days POS. The dotted line represents one healthy donor. (C) Geometric mean (GM) fluorescent intensity (MFI) values for different activation and migration markers in intermediate monocytes. The dotted line in red shows the mean of the values for the isotype controls for each marker.

In line with the cytokine data, total numbers of monocytes and neutrophils were higher at early time points than at later time points (data not shown). This was seen in all patients irrespective of symptom severity and decreased for 1 week POS in all mild patients with sequential samples. Their number was higher in P5 at the two time points tested. Because monocytes and macrophages have been shown to play an important role in pathogenesis (15), we performed a deeper phenotypic analysis of the circulating monocyte population by flow cytometry; it revealed an important increase in the population of intermediate monocytes (CD14^+^ CD16^high^ cells) detected in peripheral blood at days 2 to 3 POS (3% to 36% of cells from the live gate) (Fig. 2A and B). Intermediate monocytes expressed the highest levels of activation markers (CD86 and CD40) at 2 to 3 days POS, which was followed by the peak in the expression of differentiation and migration markers (HLA-DR, CD169, CD163, CCR-7) at 1 week POS (Fig. 2C). In summary, we observed activation of innate responses in all patients, with a clear increase in type I interferon and proinflammatory cytokines, as well as a significant increase in intermediate monocytes with activation, differentiation, and migration patterns.

### Antibody and cellular responses.

**FIG 3:**
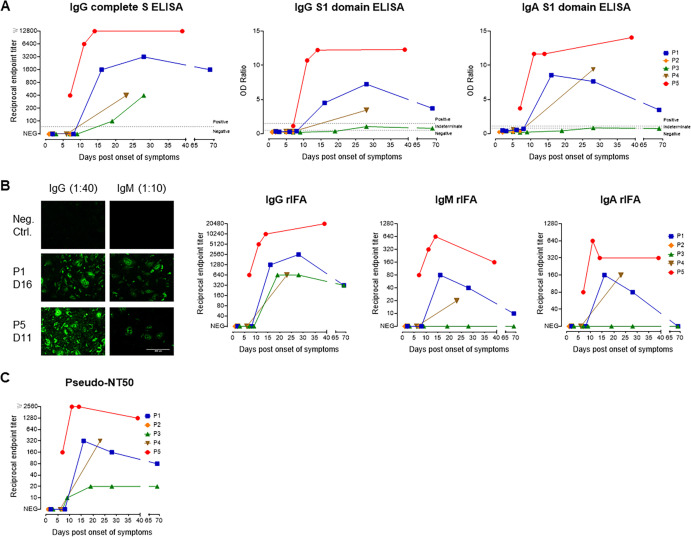
Antibody responses to SARS-CoV-2. (A) IgG and IgA antibody kinetics for P1 to -5 using ELISAs with either SARS-CoV-2 full-S (only IgG, left graph) or the S1 domain (IgG and IgA, middle and right graphs) as the antigen. For the full-S ELISA, endpoint titers were determined (starting dilution, 1:100), whereas for S1-based ELISAs, the ratio of sample to calibrator was measured (dilution, 1:100). (B) Antibody kinetics using a recombinant full-S-based immunofluorescence assay (rIFA) for IgG, IgM, and IgA isotypes using a starting dilution of 1:40 (IgG and IgA) or 1:10 (IgM). The left panel shows representative rIFA staining patterns for IgG and IgM for two patients and a negative-control serum. (C) Kinetics of neutralizing antibody endpoint titers using a pseudotyped VSV neutralization assay (starting dilution, 1:10).

Serum antibody titers against the spike protein (complete spike and S1 domain) were evaluated by enzyme-linked immunosorbent assay (ELISA) and a recombinant immunofluorescence assay (rIFA) (Fig. 3). Patients 1, 3, 4, and 5 seroconverted by 2 weeks POS, and this included P1, who had very mild and transient symptoms. For P2, no sample was available beyond day 7. P3 had no detectable IgA or IgM and only low IgG levels (Fig. 3A and B). Interestingly, this patient seroconverted only 3 weeks POS in an S1-based ELISA (Fig. 3A) but had already seroconverted after 2 weeks in complete S-based assays (rIFA and ELISA) (Fig. 3A and B) and in neutralization assays (Fig. 3C), indicating a potential role of S2 domain-specific antibodies in virus neutralization. P5 had the highest antibody levels and seroconverted earlier than patients with mild symptoms. Antibodies from all patients except P2, for whom samples from later time points were not available, were able to neutralize SARS-CoV-2 S-pseudotyped viruses; P5 again demonstrated the highest neutralizing antibody titers (Fig. 3C). For P1 and P3, a late serum sample was obtained at 69 days POS. P1 had already shown a decline in IgG, IgA, IgM, and neutralizing antibody titers (Fig. 3A to C), while the antibody levels of P3 declined only minimally (rIFA, S1 ELISA) or stayed the same (neutralization assay).

**FIG 4:**
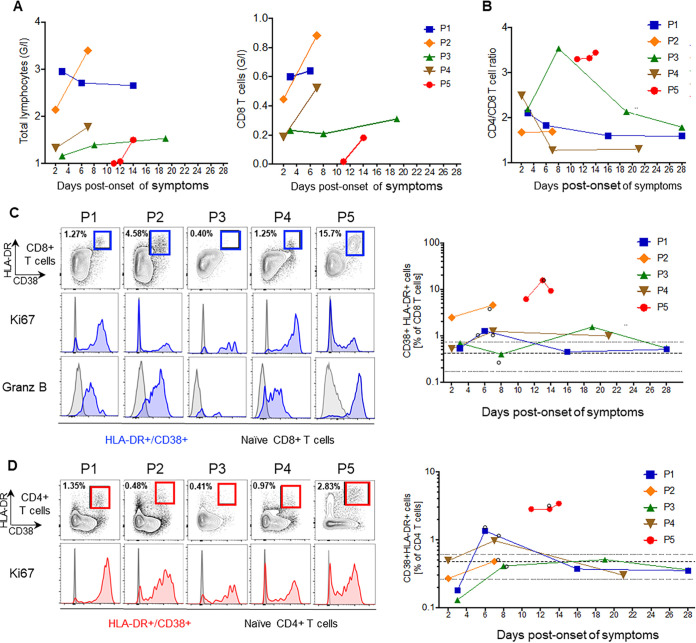
Kinetics of T-cell immune responses. (A) Graphs show the frequencies of total lymphocytes and of CD8 T cells in Giga/liter (G/l). (B) The ratio of CD4 to CD8 T cells was determined by flow cytometry as the ratio of the frequencies of CD4 and CD8 T cells (percentage of CD3 T cells). (C) Representative flow plots are shown for CD8 T-cell activation markers CD38 and HLA-DR. Histograms are shown for the expression of Ki67 (middle) and granzyme B (lower) of activated (blue) and naive (gray) CD8 T cells (gated on CD45RA^+^ CCR7^+^ CD8 T cells). The summary graph depicts the kinetics of HLA-DR^+^ CD38^+^ CD8 T cells at different time points after symptom onset. The bold dotted line is the mean of results for three healthy controls, with its range indicated as a thin dotted line; open circles identify the time points that are shown as flow plots. (D) Similar to panel C; representation of flow plots for HLA-DR^+^ CD38^+^ CD4 T cells (red), expressing the proliferation marker Ki67, with summarized data in the right graph.

We measured the frequency of T lymphocytes and detected low total lymphocyte counts, particularly of CD8^+^ T cells, at early time points; these were most pronounced in P5 (Fig. 4A and B). We further characterized the T-cell phenotype in terms of activation (expression of markers CD38 and HLA-DR), proliferation (*K_i_*-67) of CD4 and CD8 T cells, and the expression of granzyme B for CD8 T cells (Fig. 4C and D). The highest frequency of activated CD8 T cells was detected in P5; however, in this patient, very few activated cells were proliferating (Fig. 4C). Although the frequency of activated CD8 T cells was lower in patients with mild symptoms, activated CD8 T cells expressed granzyme B in all patients. For CD4 T cells, the frequency of activated cells was higher in P5 (CD38^+^ HLA-DR^+^ CD4 T cells, 2.8%) than in healthy individuals (mean, 0.49%) and even in patients with mild symptoms (CD38^+^ HLA-DR^+^ CD4 T cells, <1.35%). Activated CD4 T cells were proliferating in all infected patients (Fig. 4D). Altogether, the detection of activated and proliferative T cells in all patients suggests that even patients with mild disease are able to mount a T-cell-mediated immune response to SARS-CoV-2.

## DISCUSSION

We report the kinetics of viral loads and immune responses in the first five COVID-19 patients hospitalized in Geneva presenting with mild (P1 to -4) and severe (P5) disease. The most striking finding was a robust innate response in patients with mild symptoms characterized by early activation of monocytes. We also found prolonged viral RNA detection and measurable SARS-CoV-2-specific cellular and antibody responses despite the paucity of symptoms.

We documented high viral loads in the upper respiratory tract at diagnosis and the presence of infectious virus in both NPS and OPS, samples taken hours POS in patients presenting with mild illness. Similar results have been documented in the presymptomatic phase (16) and can partly explain the high transmissibility of SARS-CoV-2. In all patients with mild disease, infectious virus could be isolated several days after symptom onset, up to day 7. Notably in P1, symptoms had already waned for 5 days before the last successful isolation of infectious virus. We could not isolate SARS-CoV-2 in clinical specimens containing less than 1.4E6 viral RNA copies/ml, in line with other reports (17). Interestingly, in the patient with the severe disease, no virus could be isolated from the NPS during the second week of illness, possibly indicating antibody-mediated viral neutralization at that time. Except in stools and saliva, viral RNA was observed inconsistently and at low levels in other body fluids. This suggests that these fluids are not a major source of transmission. While patients with mild symptoms tend to have more limited shedding (4), prolonged RNA detection has also been reported by others (18). In our study, no infectious virus could be retrieved from such samples, arguing for lingering remnant RNA rather than for active replication. While there is a need to confirm these findings in larger cohorts, they provide important information for discharge criteria and public health decisions regarding isolation. Our data on isolation of infectious virus support most recommendations currently in effect, which advise isolation for at least 10 days POS for infection control, even in patients with very brief symptom durations.

Monocytes and macrophages play a critical role in the immunopathology of COVID-19 (15). An increased number of cytokine-producing CD14^+^ CD16^+^ monocytes and activated macrophages are seen in the blood and bronchoalveolar lavage specimens of severe patients, respectively (19–21). Zhou et al. showed that increases in activated intermediate monocytes were seen mainly in intensive-care-unit (ICU) patients as opposed to non-ICU patients (22). An interesting observation is that the rapid and drastic impact of SARS-CoV-2 infection on circulating monocytes in patients with very mild symptoms has a profile that is similar to what is reported by Zhou and colleagues for ICU patients. This new observation may be due to the fact that our study captured very early responses. The rapid mobilization of monocytes is indicative of a robust local antiviral response. In patients without complications, this early mobilization of monocytes is not sustained and rapidly declines to baseline 1 week POS. This monocyte signature is not unique to SARS-CoV-2: it has been described for dengue fever (23, 24) and other viral diseases (8, 25, 26).

In our study, all patients with mild symptoms and with samples available from day 7 POS or later mounted adaptive immune responses, leading to neutralizing antibodies. The question of the durability of the response and its quality over time remains open. As observed for P1 but not for P3, antibody titers, including neutralizing antibodies, may rapidly decline in some non- or pauci-symptomatic COVID-19 patients. Experimental studies showed that immunity against reinfection by other human CoVs wanes over a few months, enabling reinfection within the first year after acute disease (27). Low levels of nonneutralizing antibodies may enhance disease severity at the time of reexposure (28) through an antibody-mediated mechanism which has been described for other viruses but not yet observed *in vivo* for SARS-CoV-2 (29).

Our study has some limitations worth noting. Although we were able to perform a comprehensive analysis of the immune response and viral shedding, the description is limited to five patients (with early time points missing for P5 and later time points missing for P2), and our interpretation of the association between viral load and innate and adaptive responses in patients with mild COVID-19 requires confirmation with a larger sample size. Finally, we were unable to follow all patients for late time points and cannot reach a conclusion about the durability of SARS-CoV-2-specific immunity according to disease pattern.

### Conclusions.

We provide a comprehensive immunological and virological profile of the first five patients diagnosed in Geneva. Four had only mild and/or very transient symptoms, and none received immunomodulatory treatments. Importantly, we found that both in patients with mild symptoms and in those with prolonged high viral loads, as well as in one patient with severe disease, no virus could be isolated after the first week of illness.

Even in patients with mild symptoms, we found a robust innate response characterized by the mobilization of activated monocytes in the first days of infection, associated with high viral loads and the induction of interferon-dependent circulating cytokine responses. All patients mounted SARS-CoV-2-specific adaptive responses, including neutralizing antibodies.

## MATERIALS AND METHODS

### Study population and assessment.

The first five patients (P1 to P5) with COVID-19 confirmed by real-time reverse transcriptase PCR (rtRT-PCR) and quarantined at HUG were included in this single-center prospective cohort. These patients were screened for SARS-CoV-2 according to the national guidelines in place at the time, which recommended screening travelers returning from Italy and reporting respiratory and/or flu-like symptoms. Ethical approval was waived by the local ethics committee (BASEC Req-2020-00143); written informed consent for sample collection and coded data gathering was obtained from each patient. Clinical characteristics were described according to the Severe Emerging Infections clinical protocol of the International Severe Acute Respiratory and Emerging Infection Consortium (30), and disease severity was assessed according to the WHO’s classification, where mild illness corresponds to mild symptoms without pneumonia (31). If the patient agreed, we collected daily nasopharyngeal, oropharyngeal, conjunctival, sweat, and anal swabs during hospitalization, as well as saliva, urine, and stool samples (see Text S1 in the supplemental material). Plasma and serum samples were collected daily during hospitalization and at days 14 ± 2 and 28 ± 7 POS following discharge for viral load, antibody, and cytokine quantification. Whole-blood samples were used to isolate peripheral blood mononuclear cells (PBMCs) to assess cellular responses.

10.1128/mSphere.00827-20.1 TEXT S1 Detailed methods of sample collection, assessment of infectious viruses, testing for viral coinfections, high-throughput sequencing, assessment of innate immunity, references for reagents used for cell phenotyping, complete S-protein-based ELISA, recombinant immunofluorescence (rIFA) assay, and quantification of neutralizing antibodies are provided here. Download Text S1, DOCX file, 0.02 MB. Copyright © 2020 Vetter et al. 2020 Vetter et al. This content is distributed under the terms of the Creative Commons Attribution 4.0 International license.

### Virological assessment.

Quantitative rtRT-PCR was performed on all clinical samples. RNA was extracted using the NucliSens eMAG extraction kit (bioMérieux, France) and quantified with the Charité rtRT-PCR protocol (32) using *in vitro*-transcribed RNA for quantification (Project E, European Virus Archive—Global; https://www.european-virus-archive.com/). Screening for viral coinfections was performed via a large in-house panel of rtRT-PCR results (Text S1). Viral culture was performed on VeroE6 cells as previously described (33). Positive isolation of SARS-CoV-2 was confirmed upon finding the presence of a cytopathic effect (CPE) and an increase in viral RNA between two consecutive supernatant samples. NPS from P1 to P4 were tested by high-throughput sequencing (HTS) analysis in the context of SARS-CoV-2 viral genome epidemiological surveillance in Switzerland. Each sample was treated as previously published (Text S1) (34).

### Measurement of inflammatory markers and blood cell phenotyping.

Concentrations of 24 markers (Text S1) were measured in cryopreserved plasma using a Luminex assay (magnetic Luminex assay; R&D Systems) according to the supplier’s instructions. For data points below the detection limit, a value of 50% of the last standard dilution value was assumed.

For phenotyping of blood cells, cryopreserved PBMCs were thawed, counted, and divided to perform T-cell and monocyte phenotyping. T cells were stained in phosphate-buffered saline (PBS) with the LIVE/DEAD stain kit to exclude dead cells and anti-CD3, -CD4, -CD8, -CD38, and anti-HLA-DR antibodies and were then fixed and permeabilized. Anti-granzyme B and anti-Ki67 antibodies were used for intracellular staining. For phenotyping of monocytes, cells were stained with FcR binding inhibitor and anti-CD3, -HLA-DR, -CD40, -CD123, -CD169, -CD20, -CCR2, -CD14, -CD16, -CD86, -CD163, and -CCR7 antibodies. All data were acquired the same day on a Fortessa II cytometer (BD Biosciences) and analyzed using FlowJo software (v10; Tree Star).

### Antibody assays.

S1 domain-specific IgG and IgA antibody responses were measured using a commercially available kit (Euroimmun AG, Lübeck, Germany; EI 2606–9601 G and EI 2606–9601 A) according to the manufacturer's instructions. To detect antibody against the complete S protein, ELISA plates were coated with a purified trimerized S protein kindly provided by the Ecole Polytechnique Fédérale de Lausanne (EPFL). IgG, IgA, and IgM antibody titers were determined using a SARS-CoV-2 complete spike (S) protein-based rIFA (17, 35).

Neutralizing antibodies were quantified on VeroE6 cells infected with a vesicular stomatitis virus (VSV)-based SARS-CoV-2 pseudotype expressing a 19-amino-acid, C-terminally truncated spike protein (36–38) (NCBI accession no. NC_045512.2) with serially diluted sera. VeroE6 cells were infected with the virus-serum mixture, and green fluorescent protein (GFP)-positive infected cells were counted to assess titers (35). Details can be found in the supplemental material.
