# Supplementary material for: Daily Viral Kinetics and Innate and Adaptive Immune Response Assessment in COVID-19: a Case Series
Source: mSphere. 2020 Nov 11;5(6):e00827-20. doi: 10.1128/mSphere.00827-20 (PMC7657589; doi:10.1128/mSphere.00827-20)
Supplement: TABLE S1 [file mSphere.00827-20-st001.docx]

**Supplementary Table S1**

|  | **Patient 1** | **Patient 2** | **Patient 3** | **Patient 4** | **Patient 5** |
| --- | --- | --- | --- | --- | --- |
| **Smoking history** | Occasional smoker | Current smoker | No | No | No |
| **Epidemiological exposure** | Travelled to Milan Feb, 20 to Feb, 23 with patient 2 | Travelled to Milan Feb, 20 to Feb, 23 with patient 1 | Travelled to Milan Feb, 22 to Feb, 23 | Brother of patient 1 | Son returned from Singapore 30 days before symptom onset; had dinner with friends, back from Milan 12 days before symptom onset |
| **Date of illness Onset** | Feb, 25 | Feb, 27 | Feb, 26 | Feb, 28 | Feb, 27 |
| **Date of Diagnosis** | Feb, 26 | Feb, 27 | Feb, 27 | Feb, 28 | March, 4 |
|  |  |  |  |  |  |
| **Laboratory test results on hospital admission** |  |  |  |  |  |
| **Haemoglobin (g/dL)** | 167 | 167 | 140 | 159 | 132 |
| **Haematocrit (%)** | 46 | 47.2 | 38.9 | 45.9 | 40 |
| **White blood cell count (G/L)** | 7 | 10.9 | 2.8 | 6.6 | 12 |
| **Lymphocyte count (G/L)** | 2.43 | 1.99 | 0.9 | 1.46 | 0.46 |
| **Neutrophil count (G/L)** | 2.78 | 7.52 | 1.37 | 5.29 | 11 |
| **Platelets (G/L)** | 230 | 244 | 116 | 136 | 222 |
| **aPTT (sec)** | 27 |  | 27 | 25.8 | 26.8 |
| **PT (%)** | 75 | 94 | 100 | 79 | 100 |
| **INR** | 1.15 | 1.03 | 1 | 1.12 | 1 |
| **Fibrinogen (g/L)** | 2.7 | N/A | 2.4 | 3.3 | 8.4 |
| **D-dimer (ng/mL)** | N/A | N/A | 383 | N/A | N/A |
| **CRP (mg/L)** | 7.1 | 6 | 13 | 6 | 180 |
| **PCT (ug/L)** | N/A | 0.08 | N/A | 0.1 | 0.19 |
| **Sodium (mmol/L)** | 139 | 137 | 143 | 140 | 138 |
| **Potassium (mmol/L)** | 3.7 | 3.9 | 4 | 3.5 | 3.8 |
| **Urea (mmol/L)** | 4.9 | 5.2 | 5.6 | 6.8 | 4.3 |
| **Creatinine (umol/L)** | 105 | 105 | 82 | 109 | 63 |
| **eGFR (CKD-EPI) (mL/min/1.73m2)** | 83 | 82 | 92 | 82 | 98 |
| **AST (UI/L)** | 50 | 39 | 26 | 25 | 24 |
| **ALT (UI/L)** | 66 | 58 | 27 | 36 | 16 |
| **Phosphatase alcaline (UI/L)** | 104 | 44 | 56 | 70 | 49 |
| **GGT (UI/L)** | 46 | 105 | 18 | 36 | 38 |
| **Total bilirubin (umol/L)** | 7 | 7 | 5 | 8 | 9 |
| **Other** | *Chlamydophila/Mycoplasma* (Throat swab) PCR neg | Blood culture on admission: no pathogen growth | Urine culture: no pathogen growth | Blood culture on admission: no pathogen growth | Blood culture on admission: no pathogen growth Urinary *Legionella pneumophila* and *Streptococcus pneumoniae* Ag negative Chlamydophila/Mycoplasma (Throat swab) PCR neg Urine culture: no pathogen growth  Skin biopsy : suprabasal acantholysis, swollen multinuclear cells, lymphohistiocytic infiltrate of the dermis |
| **Viral co-infection** | Adenovirus | None detected | None detected | None detected | None detected |
| **Imaging** |  |  |  |  |  |
| **Chest X-ray** | N/A | N/A | N/A | No infiltrates | Bilateral patchy infiltrates |
| **CT-Scan** | N/A | N/A | N/A | N/A | Left posterior basal consolidation, bilateral patchy infiltrates |
|  |  |  |  |  |  |
