# Supplementary figures and images for: Daily Viral Kinetics and Innate and Adaptive Immune Response Assessment in COVID-19: a Case Series
Source: mSphere. 2020 Nov 11;5(6):e00827-20. doi: 10.1128/mSphere.00827-20 (PMC7657589; doi:10.1128/mSphere.00827-20)

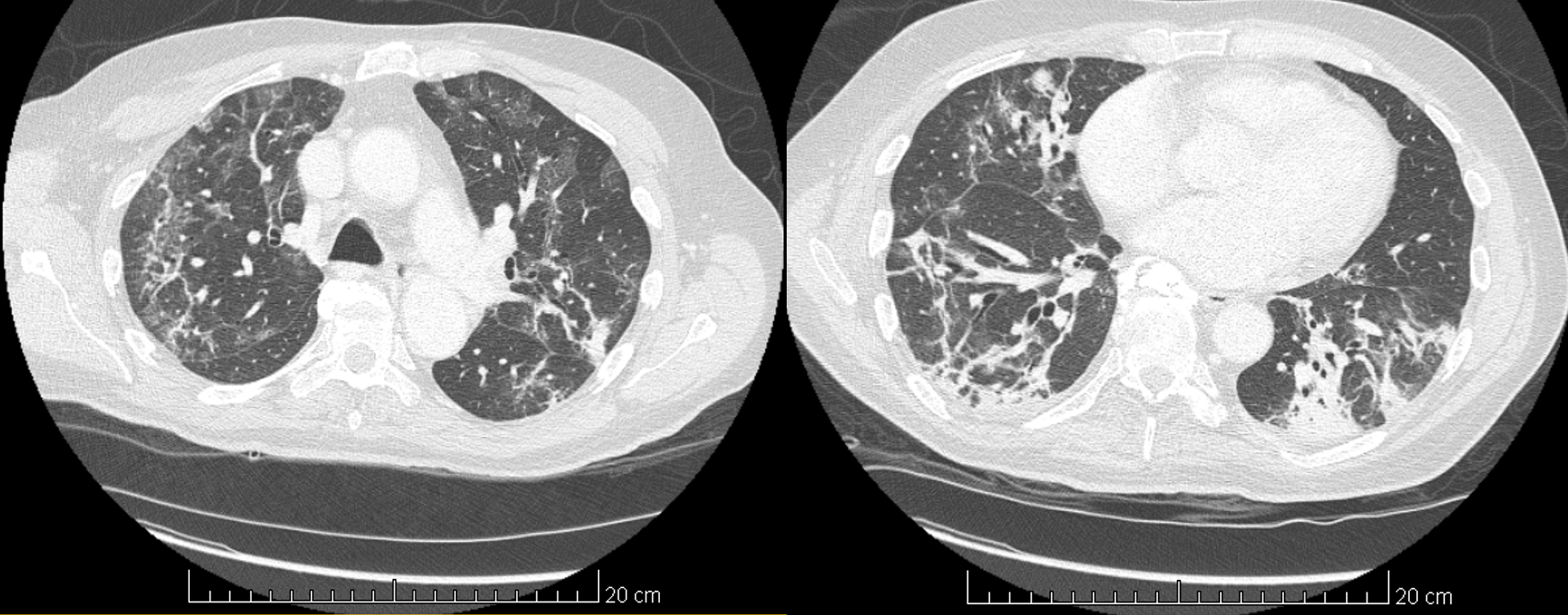

Supplement: FIG S1 [file mSphere.00827-20-sf001.tif]

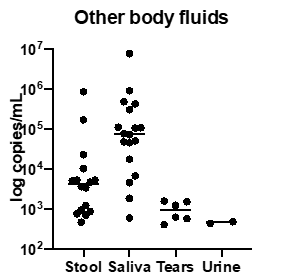

Supplement: FIG S2 [file mSphere.00827-20-sf002.tif]

**Supplementary Figure S3**

**A)**


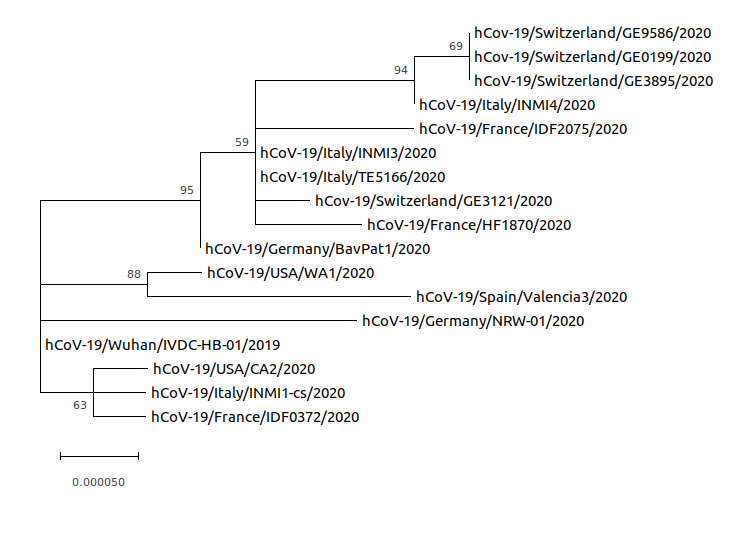


**B)**

Supplement: FIG S3 [file mSphere.00827-20-sf003.docx]
