# Supplementary material for: Daily Viral Kinetics and Innate and Adaptive Immune Response Assessment in COVID-19: a Case Series
Source: mSphere. 2020 Nov 11;5(6):e00827-20. doi: 10.1128/mSphere.00827-20 (PMC7657589; doi:10.1128/mSphere.00827-20)
Supplement: TEXT S1 [file mSphere.00827-20-s0001.docx]

**Supplementary Methods**

Sample collection

We collected daily nasopharyngeal, oropharyngeal, conjunctival, sweat and anal swabs as well as saliva, urine and stool samples if the patient agreed, using 3mL universal transport medium tubes (Copan, Brescia, Italy). Urine and stools were collected daily (when available) in plastic tubes without additives. Plasma and serum were collected in EDTA and in SST II plastic tubes, daily during hospitalization and at days 14 +/- 2 and 28 +/- 7 post onset of symptoms (POS) following discharge for viral load, antibody (Ab) and cytokine quantification. Cell-preparation tubes (CPT) with sodium citrate were used for collection of whole blood and separation of peripheral blood mononuclear cells (PBMC) to assess cellular responses.

Assessment of infectious viruses

VeroE6 cells were seeded at a density of 8x104 cells/well in a 24-well plate and inoculated with 200μl of viral transport medium the following day. Cells were inoculated for 1h at 37°C, then inoculum was removed, cells were washed 1x with PBS and then regular cell growth medium containing 10% FCS was added. Cells were observed on days 2, 4, and 6 for the presence of CPE by light microscopy. Supernatant was harvested upon the first observation of a CPE, or, if no CPE was observed, at the end of the experiment on day 6.

Testing for viral co-infections

Nasopharyngeal swabs were screened by acid nucleic detection for the presence of influenza A and B virus, respiratory syncytial virus (RSV) A and B, parainfluenza 1 to 4, human metapneumovirus, rhinoviruses, enteroviruses, bocavirus 1, adenovirus and human coronaviruses 229E, OC43, HKU1 and NL63 using either an in-house RT-PCR panel or the multiplex RT-PCR Fast-Track Diagnostics Resp21 commercial panel (Fast-Track Diagnostics, Esch-sur-Alzette, Luxembourg).​

High-throughput sequencing

110 µl of NPS were centrifuged at 10,000 × g for 10min. One-hundred μl of cell-free supernatant were treated with 20 U of Turbo DNAse (Ambion, Rotkreuz, Switzerland). Viral nucleic acids were extracted with TRIzol (Invitrogen, Carlsbad, CA, USA) and re-suspended in 10 μl of RNAse-free water. Ribosomal RNA was removed using the Ribo-Zero Gold depletion kit (Illumina, San Diego, US). Thereafter, libraries were generated using the TruSeq total RNA preparation protocol (Illumina) with dual indexing and loaded on the HiSeq 4000 platform (Illumina) using the 2x100-bp protocol. Raw data were analyzed as follows: duplicate reads were removed using cd-hit (v4.6.8). Then reads were then trimmed to remove low-quality and adapter sequences using Trimmomatic (v0.33). Next reads were mapped against the reference sequences MN908947.3 using the SNAP nucleotide aligner program. (2) The four hCoV-19 complete sequences hCoV-19/Switzerland/GE3895/2020 (P1), hCoV-19/Switzerland/GE9586/2020 (P2), hCoV-19/Switzerland/GE3121/2020 (P3), and hCoV-19/Switzerland/GE0199/2020 (P4) were all submitted and made available via GISAID.

Assessment of innate immunity

The following list of markers were tested in Luminex : CD40L, GM-CSF, Granzyme B, IFN-a, IFN-g, IL1a, IL1-b, IL1R-a, IL-2, IL-4, IL-6, IL-8, IL-10, IL-12p70, IL-13, IL-15, IL17A, IL-33, IP-10, MCP-1, MIP-1a, MIP-1b, PD-L1, TNF-a. The mean fluorescence intensity of each marker was read on the Bio-Plex 200 array reader (Bio-Rad Laboratories) using the Luminex xMAP Technology (Luminex Corporation). Sample concentrations were calculated using a five-parameter logistic regression curve (Bio-Plex Manager 6.0)

References for reagents used for cell phenotyping

LIVE/DEAD™ Fixable Aqua Dead Cell Stain Kit (Life Technologies), Fixation/Permeabilization kit (Invitrogen 00-8333-56), anti-CD3 (clone SK7 BioLegend), anti-CD4 (clone RPA-T4, BD), anti-CD8 (clone SK1, BD), CD38 (clone HIT2, BioLegend), HLA-DR (clone L243, BioLegend), Granzyme B (clone GB11, BD), Ki67 (clone B56, BD), FcR binding inhibitor (14-9161-73, Invitrogen), anti-CD3 (clone SP34-2, BD), HLA-DR (clone G46-6, BD), CD40 (clone 5C3, BD), CD123 (clone 7G3, BD), CD169 (clone 7-239, Biolegend), CD20 (clone, BD ), CCR2 (clone REA264, Miltenyi), CD14 (clone M5E2, BD), CD16 (clone 3G8, BD), CD86 (clone IT2.2 Biolegend), CD163 (clone GHI/61, BD), CCR7 (clone 3D12, BD)

Complete S protein-based ELISA

We coated 37.5ng/well of a purified trimerized S protein diluted in 0.1M sodium carbonate buffer pH 9.6 O/N at 4°C. Plates were blocked (5% milk PBS-T) and 100μl of 2-fold serially diluted sera (range 100-12,800) were applied to wells with and without antigen. After incubation (1h at 37°C) and washing (3x 1min with PBS-T) 100μl HRP-conjugated anti-human IgG (Jackson Immunoresearch, #109-036-098) diluted 1:16,000 was added. After incubation plates were washed and 100μl of TMB substrate (Invitrogen) was added for 10min. The reaction was stopped by 100μl of 1N HCl and plates were read at 450nm. OD450 values of blank wells were subtracted from values of antigen containing wells for each serum and each dilution. The cut-off to determine the ELISA titer was set at 0.29 OD450, just before OD values reach the plateau phase.

rIFA assay

Briefly, pCG1 vector expressing SARS-CoV-2 S protein (kindly provided by M. Hoffmann and S. Pöhlmann, DPZ Göttingen) was transfected into Vero B4 cells using Fugene HD (Promega #E2311), spotted on multitest microscopy slides (DUNN Labortechnik GmbH #40-412-05) and fixed with ice-cold Acetone/Methanol (1:1). To perform the rIFA sera were inactivated for 30min at 56°C. To remove IgG antibodies for determination of IgM antibody titers, sera were treated with Eurosorb reagent (Euroimmun AG #1270-0145). Slides were rehydrated in PBS-T for 5min and blocked (5% milk PBS-T) for 30min at room temperature. Sera were diluted using a starting dilution of 1:40 for IgG/IgA and 1:10 for IgM and 30 μl were applied to each spot. After incubation (1h, 37°C) slides were washed (3x1min, PBS-T) and 25μl of Alexa488-conjugated goat anti-human-IgG, -IgM. or -IgA antibody (Jackson ImmunoResearch #109-036-098, #, #) diluted 1:200 in PBS was applied. After incubation (37°C, 45min), slides were washed as before and briefly rinsed with dH_2_O before mounting with glycerol.

Quantification of neutralizing antibodies

VeroE6 cells were seeded in 96-well plates at 2 x 10^4^ cells per well and grown into confluent monolayer overnight. Sera from patients were inactivated at 56°C for 30 minutes and diluted from 1:5 to 1:1280 in DMEM 2% FBS. VSV-based SARS-CoV-2 pseudo-types (3, 4) expressing a 19 amino acid C-terminal truncated spike protein (5) (NCBI Reference sequence: NC_045512.2) were diluted in DMEM 2% FCS in order to have MOI=0.01 per well and added on top of serum dilutions (final serum dilutions obtained were from 1:10 to 1:2560). The virus-serum mix was incubated at 37°C, for 2h. Vero E6 were then infected with 100µl of virus-serum mixtures. After incubation at 37°C for 1.5h, cells were washed once with 1X PBS and DMEM 10% FBS was added. After 16-20h of incubation at 37°C in 5% CO_2_, cells were fixed with 4% formaldehyde solution for 15min at 37°C and nuclei stained with 1µg/ml DAPI solution. GFP positive infected cells were counted with ImageXpress® Micro Widefield High Content Screening System (Molecular Devices) and data analyzed with MetaXpress 5.1.0.41 software.

**REFERENCES**

1. Ambrosioni J, Bridevaux PO, Wagner G, Mamin A, Kaiser L. Epidemiology of viral respiratory infections in a tertiary care centre in the era of molecular diagnosis, Geneva, Switzerland, 2011-2012. Clin Microbiol Infect. 2014;20(9):O578-84.

2. Zaharia M, Bolosky W, Curtis K, Fox A, Patterson D, Shenker S, et al. Faster and More Accurate Sequence Alignment with SNAP. arXiv:11115572 2011.

3. Berger Rentsch M, Zimmer G. A vesicular stomatitis virus replicon-based bioassay for the rapid and sensitive determination of multi-species type I interferon. PloS one. 2011;6(10):e25858.

4. Torriani G, Trofimenko E, Mayor J, Fedeli C, Moreno H, Michel S, et al. Identification of Clotrimazole Derivatives as Specific Inhibitors of Arenavirus Fusion. Journal of virology. 2019;93(6).

5. Fukushi S, Mizutani T, Saijo M, Matsuyama S, Miyajima N, Taguchi F, et al. Vesicular stomatitis virus pseudotyped with severe acute respiratory syndrome coronavirus spike protein. The Journal of general virology. 2005;86(Pt 8):2269-74.
